# Galectins and galectin-mediated autophagy regulation: new insights into targeted cancer therapy

**DOI:** 10.1186/s40364-023-00466-9

**Published:** 2023-02-22

**Authors:** Dan Liu, Hongtao Zhu, Chuanzhou Li

**Affiliations:** 1 Department of Medical Genetics, School of Basic Medicine, Tongji Medical College, Huazhong University of Science and Technology, Wuhan, China; 2 Department of Neurosurgery, Tongji Hospital, Tongji Medical College, Huazhong University of Science and Technology, Wuhan, China

**Keywords:** Galectin, Autophagy, Cancer therapy, Clinical trials

## Abstract

Galectins are animal lectins with specific affinity for galactosides via the conserved carbohydrate recognition domains. Increasing studies recently have identified critical roles of galectin family members in tumor progression. Abnormal expression of galectins contributes to the proliferation, metastasis, epithelial-mesenchymal transformation (EMT), immunosuppression, radio-resistance and chemoresistance in various cancers, which has attracted cumulative clinical interest in galectin-based cancer treatment. Galectin family members have been reported to participate in autophagy regulation under physiological conditions and in non-tumoral diseases, and implication of galectins in multiple processes of carcinogenesis also involves regulation of autophagy, however, the relationship between galectins, autophagy and cancer remains largely unclear. In this review, we introduce the structure and function of galectins at the molecular level, summarize their engagements in autophagy and cancer progression, and also highlight the regulation of autophagy by galectins in cancer as well as the therapeutic potentials of galectin and autophagy-based strategies. Elaborating on the mechanism of galectin-regulated autophagy in cancers will accelerate the exploitation of galectins-autophagy targeted therapies in treatment for cancer.

## Background

Galectins (Gals), first characterized in the mid-1970s [1], were initially referred to as S-type lectins due to their sulfhydryl dependence and were then defined as galectins in 1994 [2]. Galectins possess multiple functions, including mediating cell–cell and cell–matrix adhesion, regulating cell growth, apoptosis, pre-mRNA splicing, signal transduction, and immune regulation [3]. They are widely expressed in various cell types and are renowned for their capacity to increase cancer cell invasiveness and resistance to chemotherapy [4]. Alterations in galectin expression are closely related to cancer biology including vascular formation, cell migration, and tumor immune evasion during carcinogenesis [4]. In most cases, the upregulated expression of galectin in the tumor microenvironment predicts a poor prognosis [5]. Therefore, galectins have drawn particular attention in cancer research and therapy.

Notably, galectins are recently shown to be associated with autophagy regulation in different cancers. For decades, autophagy, a natural and conserved degradation form of cell, has been widely reported to maintain intracellular homeostasis under physiological and pathological conditions, and dysregulation of autophagy is associated with a variety of diseases (e.g., cancer, neurodegenerative diseases, type II diabetes, and heart disease) [6, 7]. Particularly in cancer, autophagy not only suppresses tumors by removing damaged organelles and restricting cell proliferation by destabilizing the genome, but also facilitates tumor growth by satisfying the metabolic demands of cancer cells and inducing chemoresistance, promoting cross-talk between tumor cell and stroma, especially in nutrient-limited microenvironments [8, 9]. Although considerable numbers of autophagy-related genes have now been discovered, the mechanisms of autophagy-related signaling and how these pathways impact cancer biology are still controversial due to that the roles of autophagy are largely depending on the tumorigenesis stage and the tumor type.

The association of galectins and autophagy in carcinogenesis has been evidenced by growing studies showing that the aberrant expression of galectins enhances the dependence of cancer cells on autophagy. It is believed that inhibiting autophagy and galectins activities in specific types of tumors may serve as useful supplement to current therapies. In this review, we enumerate the current understanding of galectins and autophagy in cancer, summarize the recent advances in potential therapeutic approaches related to galectin and autophagy regulation in cancer, with particular focus on the current use of galectin inhibitors and autophagy modulators in cancer therapy. Lastly, we prospect the challenges and therapeutic opportunities of galectin and autophagy targeted strategies in future clinical applications.

### Overview of galectins

#### Galectin structure and classification

**Fig. 1:**
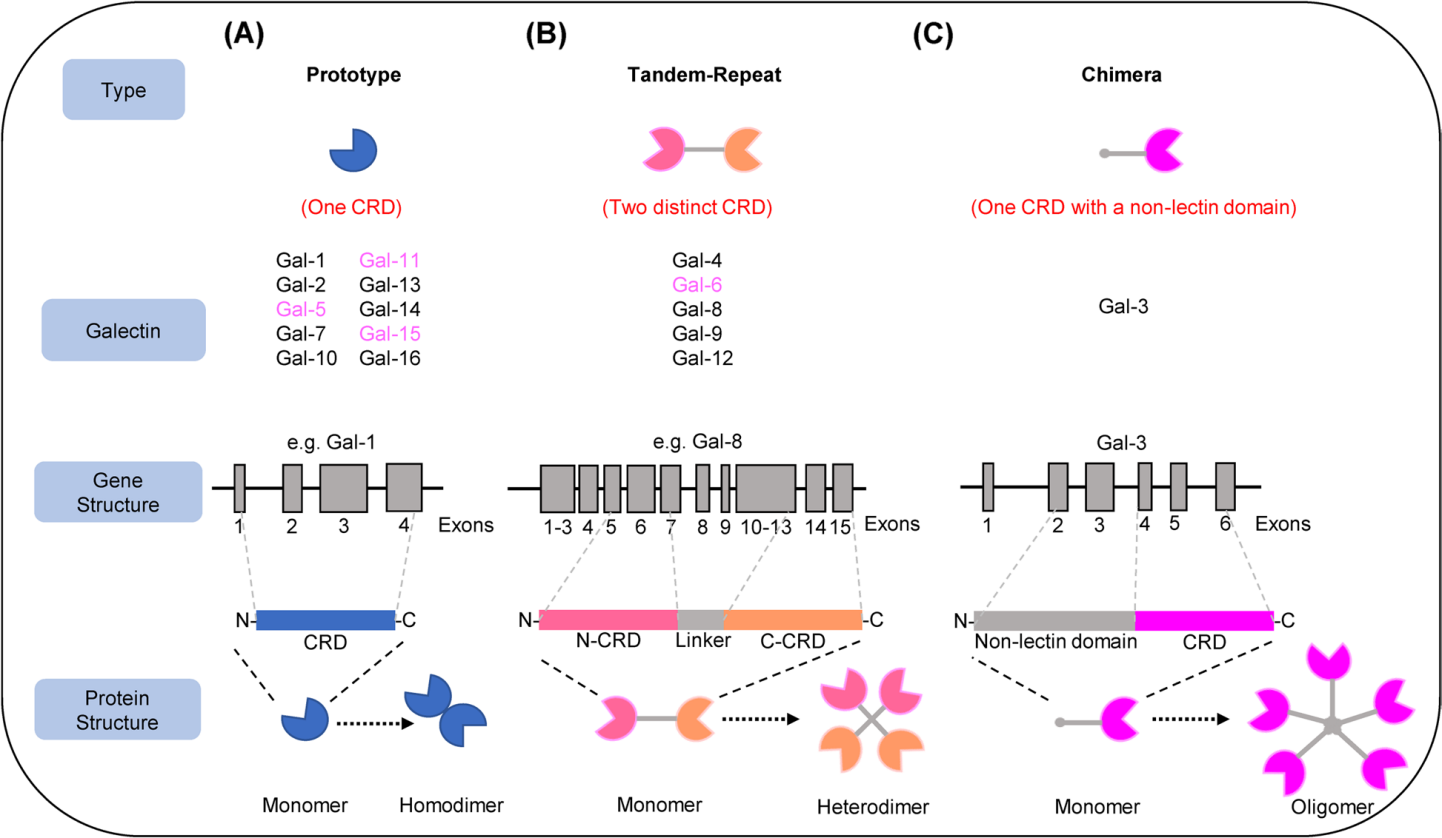
Gene and protein structures of galectins. **A** Prototype galectins that contain a CRD capable of forming monomers or non-covalent homodimers. **B** Tandem repeat galectins. Two different but homologous CRDs connected by a functional linker peptide, present in monomeric or oligomeric form. **C** Chimera galectins consist of an approximate 120-amino acid N-terminus attached to a CRD and form oligomers with increased binding affinity. Members of galectin families not discovered in humans are marked in purple. Representative gene structures are illustrated as indicated

Galectins are a class of glycan-binding proteins consisting of one or two conserved carbohydrate recognition domains (CRDs) containing approximately 130 amino acids [10]. Galectin homologues are highly conserved in primitive organisms such as sponges and nematodes. There are 16 different galectins identified in mammals, and 12 of them have been founded in humans except galectin-5, -6, -11 and -15 (Table 1) [11]. Galectins are often divided into three subgroups based on their distinct biochemical structural characteristics: prototypical galectins have one CRD per subunit and may present as noncovalently linked homodimers (galectin-1, -2, -5, -7, -10, -11, -13, -14,-15 and -16); the tandem-repeat type galectins contain two homologous CRDs that are joined by a functional linker peptide (galectin-4, -6, -8, -9 and -12); the chimera type galectin (galectin-3) contains a non-lectin N-terminal region connected to a CRD [4, 12] (Fig. 1.). Each galectin has an individual carbohydrate binding preference, due to its bivalent or multivalent interactions with glycans [13]. It is noteworthy that not all galectins have the ability to bind to glycans. For example, Gal-10 can bind to eosinophilic granule cationic ribonucleases without the help of carbohydrates [14], and Gal-13 does not bind to β-galactosides and forms dimers via intermolecular disulfide bridges [15].

**Table 1:** Galectin members and tissue-specific distribution in humans

	Gene name	Human Chromosomal location	Molecular Weight (kDa)	Tissue distribution in human	Refs
Gal-1	*LGALS1*	22q12	14–15	Expressed in many tissues and cell types	[16]
Gal-2	*LGALS2*	22q12	14	Gastrointestinal tract, placenta,	[16]
Gal-3	*LGALS3*	14q21-22	29–35	Expressed in many tissues and cell types, especially in immune and epithelial cells	[16]
Gal-4	*LGALS4*	19q13.2	37	Mostly expressed in the gastrointestinal tract of animals, hippocampal and cortical neurons	[10]
Gal-7	*LGALS7*	19q13.2	15	Stratified epithelia, skin, fetal heart, gastrointestinal tract	[10, 17]
Gal-8	*LGALS8*	1q42.11	35	Brain, liver, kidney, heart, lung, spleen and hind limb	[10]
Gal-9	*LGALS9*	17p11.2	36	Skin and gastrointestinal epithelial cells, liver, thymus	[11, 17]
Gal-10	*LGALS10*	19q13.1	16.5	Eosinophils	[10]
Gal-12	*LGALS12*	11q13	35	Adipose tissue	[10]
Gal-13	*LGALS13*	19q13.1	16	Placenta	[10]
Gal-14	*LGALS14*	19q13.2	18	Eosinophils, placenta	[18]
Gal-16	*LGALS16*	19q13.2	16	Placenta	[19]

*LGALS* Lectin, Galactoside-Binding, Soluble

#### Cellular functions of galectin

As a soluble protein, galectin plays key regulatory roles both inside and outside of the cell. Intracellular galectins exhibit cellular functions such as regulation of cell growth and apoptosis in a carbohydrate-independent manner and modulate intracellular signaling pathways through protein–protein interactions with other cytoplasmic and nuclear proteins [20]. Although lacking the typical secretory signaling peptides, galectins can be secreted to extracellular space through non-classical secretory pathways. For instance, Epstein-Barr virus (EBV) infected nasopharyngeal carcinoma cells release Gal-9 via exosomes [21]. Secreted galectins can regulate cell adhesion, migration and invasion by recognizing and binding to carbohydrates of glycoproteins or glycolipids [3]. In tumor cells, the function of galectins relates to their subcellular localization. The intracellular galectins enhance oncogenic signaling and reduce apoptosis, promote proliferation of tumor cells [22], while the extracellular galectins, on the other hand, bind to glycoconjugate ligands on surrounding cells and finally regulate the immune responses and tumor growth [23, 24].

### Role of galectin in tumor progression

Studies have shown that galectins play vital roles in multiple processes (e.g. tumorigenesis, metastasis and angiogenesis) of tumor progression. Interestingly, galectins possess diverse functions in different tumors (Table 2) and regulate tumor progression through distinct mechanisms.

**Table 2:** Functions of galectins in different tumors

Galectin (Gal)	Cancer type	Effect	Mechanisms	Refs
Gal-1	Glioma, breast, ovarian, cervical, pancreas, prostate, thyroid, colorectal, lung, melanoma, neuroblastoma, hepatic, gastric, bladder	Increases cell growth, migration, invasion, angiogenesis, metastasis and chemotherapy resistance, induces tumor immune evasion, promotes tumor progression, inhibits apoptosis	Interacts with glycoconjugates and regulates the biological activities of H-Ras/MEK/ERK, β-catenin pathways in the tumor microenvironment	[16, 24–29]
Gal-3	Glioma, breast, ovarian, cervical, pancreas, prostate, thyroid, colorectal, liver, lung, melanoma, neuroblastoma, bladder, gastric, tongue, renal	Increases metastasis formation, reduces apoptosis and induces tumor immune evasion, increase adhesion, tumor growth and motility, induces chemoresistance, cell proliferation, angiogenesis and migration, regulate cell cycle and cell transformation	Regulates M2 polarization of macrophages and triggers apoptosis via its binding to antitumor T cells. Induces the expression of matrix metalloproteinases through p38-MAPK pathway. Maintains mitochondrial homeostasis and regulates tumor metabolism	[17, 30–45]
Gal-8	Glioma, breast, ovarian, prostate, thyroid, colon, liver, lung, bladder, renal, laryngeal	Mediates cell adhesion, migration, proliferation and survival	Binds to integrins and activates the downstream FAK pathway, interacts with activated leukocyte cell adhesion molecule receptors	[46–57]
Gal-9	Glioma, breast, ovarian, cervical, pancreas, prostate, colon, liver, lung, melanoma, renal, oral, myeloma, cholangiocarcinoma, esophageal	Affects cell adhesion and metastasis, induces apoptosis	Regulates JNK and p38 MAP kinase pathways, mitochondrial pathway, blocks adhesion to endothelium and extracellular matrices	[58–68]
Gal-4	Glioma, breast, ovarian, cervical, pancreas, colorectal, liver, lung	Promotes adhesion, reduces cell migration and metastasis formation, induces cell cycle arrest	Interacts with and down-regulates the functions of Wnt signaling pathway. Interferes with the integrin beta 4/Src/FAK cascade	[69–76]
Gal-7	Breast, ovarian, colon, cervical, neuroblastoma, melanoma, gastric, urothelial, thyroid	Correlates with cell proliferation, migration, infiltration and chemosensitivity	Inhibits the TGF beta/Smad3 pathway	[77–80]
Gal-2	Breast, colon	Increases adhesion	Unclear	[81, 82]
Gal-12	Cervical, colorectal	Reduces cell growth	Binds to SLC1A5 and inhibits glutamine anaplerosis	[83]

#### Galectin-1 (Gal-1)

Gal-1 was the first identified member in the galectin family, and the carbohydrate-binding action of Gal-1 occurs extracellularly while the biological functions of Gal-1 depend on its cellular localization and local concentration [24]. The dysregulated expression of Gal-1 in tumors has been widely reported and it is considered as a biomarker for malignant progression in urothelial urinary bladder carcinoma and carcinoma of the aerodigestive tract [25–27]. Gal-1 is expressed and secreted by numerous tumors such as gastric, hepatocellular, pancreatic, colorectal, prostate, neuroblastoma, glioma, aggressive melanoma, osteosarcoma, breast, lung, bladder, thyroid, ovarian epithelial, myeloma, head and neck, cervical, and endometrial cancers [16, 28]. Elevated Gal-1 expression in tumor correlates with multiple processes including cell cycle regulation, apoptosis, cell adhesion, migration, angiogenesis, drug resistance and immunosuppression by interacting with glycoconjugates and regulating the biological activities of H-Ras/MEK/ERK, β-catenin pathways in the tumor microenvironment [5, 28]. The role of Gal-1 in mediating tumor growth and metastasis has also been verified in tumor-bearing animal models, which further indicates that inhibition of Gal-1 may serve as a promising strategy for cancer therapy [29].

#### Galectin-3 (Gal-3)

Gal-3, initially named CBP35 in 1983, is the only chimeric galectin discovered in vertebrates as well as one of the most intensively studied galectins [30]. In addition to various biological functions including neurogenesis and inflammation under physiological conditions, Gal-3 has been reported to be associated with cancer cell proliferation, migration, invasion, angiogenesis and immunosuppression through mechanisms that Gal-3 regulates M2 polarization of macrophages, triggers apoptosis by binding to antitumor T cells, induces the expression of matrix metalloproteinases through p38-MAPK pathway, maintains mitochondrial homeostasis and regulates tumor metabolism. [16, 31]. Gal-3 in the cytoplasm is involved in the regulation of many cancer-related signaling pathways involving oncogenes such as RAS, BCL-2 and MYC [32–34]. In addition, intracellular Gal-3 can inhibit the mitochondrial apoptotic pathway induced by cisplatin or etoposide in prostate and breast cancer cells [32, 35]. Given that inhibition of Gal-3 may be crucial to increase the sensitivity of tumor cells to chemotherapy, the role of Gal-3 in chemoresistance is also of interest [36]. Recent studies suggest that upregulation of Gal-3 is positively correlated with tumor progression and metastasis in a variety of cancers, including breast cancer, glioma, thyroid cancer, gastric cancer, lung cancer, kidney cancer, prostate cancer, melanoma, ovarian cancer, cervical cancer, bladder cancer and pancreatic cancer [16, 17, 37–39]. In addition to tumor cells, the tumor microenvironment induces Gal-3 expression to maintain cellular homeostasis and promote tumor growth as well [40]. For example, Gal-3 is enriched in hypoxic regions and promotes malignancy under hypoxia in glioblastoma, breast cancer and non-small cell lung cancer [41, 42]. Notably, Gal-3 in the tumor microenvironment has distinct roles when located intracellularly and extracellularly [43]. When accumulated in the nucleus, Gal-3 shows anticancer action; but it promotes tumor growth when expressed in the cytoplasm in tongue cancer and prostate cancer cells [44, 45].

#### Galectin-8 (Gal-8)

Hadari and colleagues in 1995 first identified the ‘tandem repeat’-type galectin Gal-8 [46], which was then isolated from the prostate [PCTA-1] and the lung Po66 carbohydrate-binding protein (Po66-CBP) [47, 48]. Gal-8 is widely expressed in normal tissues, whereas increased expression is present in breast cancer, laryngeal cancer and cutaneous lymphoma, with decreased expression in skin cancer and several gastrointestinal cancers including pancreatic, liver and colon cancers [49–53]. Intriguingly, Gal-8 is expressed only in tumor prostate cells other than normal prostate tissue, implying its potential as a specific biomarker for prostate cancer [54]. Gal-8 has also been reported to involved in tumor cell proliferation, adhesion, migration and EMT in lung cancer, breast cancer, ovarian cancer, and malignant glioma [17, 38, 55–57]. Mechanically, Gal-8 regulates the adhesion and migration of lung cancer cells by binding to integrins and activates the downstream focal adhesion kinase (FAK) pathway [56], promoting breast cancer cell migration through interacting with activated leukocyte cell adhesion molecule such as CD166/ALCAM receptors [57].

#### Galectin-9 (Gal-9)

Gal-9 is of particular interest due to its multiple biological functions and potent immunomodulatory effects. In 1997, Gal-9 was initially isolated from mouse embryonic kidney [58], and it was found to be an autoantigen linked to Hodgkin's lymphoma and a novel eosinophil chemotactic agent produced by T cells [59, 60]. Gal-9 was highly expressed in Hodgkin's lymphoma, colorectal, oral and pancreatic cancers, whereas expressed at a low level in breast cancer, liver cancer, lung cancer, prostate cancer, renal cancer and melanoma [16]. In solid tumors, Gal-9 expression is closely related to cell proliferation and metastasis via JNK and p38 MAP kinase pathways, mitochondrial pathway, blockade of adhesion to the endothelium and extracellular matrixes. Gal-9 can induces apoptosis in esophageal cancer cells, myeloma cells, bile duct cancer cells and ovarian cancer cells [61–64]. In breast cancer and malignant melanoma, overexpression of cytoplasmic Gal-9 may inhibit tumor metastasis and attenuate recurrence [65]. More recently, Gal-9 indicates emerging potential as a therapeutic target for cancers, by serving as a good prognostic factor for kidney cancer and an independent indicator of poor prognosis in glioma [38, 66]. Gal-9 also possesses immunomodulatory effects by serving as a ligand for T-cell immunoglobulin and mucin structural domain protein 3 (Tim-3), which are crucial for anticancer immunity [67]. Notably, combined inhibition of Gal-9 and immune checkpoint protein programmed cell death ligand 1 (PD-L1) in glioblastoma is expected to overcome the failure of mono-target immunotherapy [68].

#### Other galectins

Few studies have focused on the phenotype and function of altered Gal-2 expression in cancers versus normal tissues. Increased circulating levels of Gal-2 have been reported in the serum of patients with colon and breast cancers [81]. Encouragingly, Gal-2 was recently identified as an immunotherapeutic target for triple-negative breast cancer using an in vivo clustered regularly interspaced short palindromic repeats (CRISPR)-based screening [82].

Gal-4 was first found in porcine oral epithelium and rat intestine [69, 70]. Despite abundant expression in the luminal epithelium of the gastrointestinal tract [71], Gal-4 was found upregulated in the corresponding cancerous tissues, such as the liver, mammary gland, ovary, and brain [72]. Recent data showed that Gal-4 was highly enriched in the serum of patients with cervical cancer [73]. Interestingly, Gal-4 has tumor suppressive effects in colorectal and pancreatic adenocarcinomas [74, 75], whereas it is an unfavorable prognosis factor in patients with lung adenocarcinomas [76]. Moreover, Gal-4 interacts with and down-regulates the functions of Wnt signaling pathway and interferes with the integrin β4/Src/FAK cascade.

Gal-7 is a transcriptional target of the tumor suppressor protein p53 and was initially identified by Magnaldo et al. in 1995 [77]. Gal-7 is highly expressed in breast cancer, thyroid cancer and pharynx cancer, however, its expression is lower in stomach cancer, colon cancer, cervical squamous cancer and uroepithelial bladder cancer [16]. Gal-7 can serve as a prognostic marker for epithelial ovarian cancer [78]. Moreover, Gal-7 is a negative growth regulator in neuroblastoma and may predict chemoresistance in pancreatic cancer [79, 80].

Other mammalian galectins including Gal-10, Gal-12, Gal-13, Gal-14, and Gal-16 were relatively less investigated in cancers. Gal-10 was reported to be associated with colon cancer and gastric cancer [84, 85]. Down-regulation of Gal-12 is observed in colon cancer with a tumor suppressive function by binding to SLC1A5 and suppressing glutamine anaplerosis [83]. Findings regarding Gal-13, Gal-14 and Gal-16 are mainly limited to placental tissues. It is worth noting that, despite the lack of studies including normal tissues as control, these three galectins are believed to participate in cancer biology [18, 19]. In conclusion, these findings together strongly support the essential roles of galectin family members in carcinogenesis, further highlighting the basis for galectin-targeted cancer therapy as demonstrated below.

### Targeting galectin for cancer therapy

The development of galectin inhibitors started decades ago when the essential roles of galectins in tumor growth were initially discovered. So far, two major groups of galectin inhibitors are available: carbohydrate-based and non-carbohydrate inhibitors [86]. Carbohydrate inhibitors include glycodimers and modified glycans such as inhibitors based on galactose, talose, lactose, N-acetylamino lactose, and thiogalactoside [87]; non-carbohydrate inhibitors include peptides, peptidomimetic inhibitors and heterocyclic compounds [86]. Currently, typical inhibitors targeting Gal-1 (Table 3) and Gal-3 (Table 4) have already shown significant potential in clinical applications of cancer therapy.

**Table 3:** Gal-1 inhibitors in tumor progression

Inhibitor	Target	Effect	Refs
Thiodigalactoside (TDG)	Melanoma, colon and breast cancer xenografts	Inhibition of angiogenesis and tumor growth, reduction of lung metastases, induction of apoptosis	[88]
OTX008	Ovarian cancer xenografts, head and neck squamous cell carcinomas, oral squamous cell carcinoma, thyroid cancer	Inhibition of tumor growth, angiogenesis, proliferation, invasion and migration	[89]
Anginex (β-pep25)	Ovarian and breast cancer xenografts	Inhibition of tumor growth, proliferation and angiogenesis	[90]
6DBF7; DB16; DB21	Lung and ovarian cancer and melanoma xenografts	Inhibition of tumor growth and angiogenesis	[86]
GM-CT-01 (DAVANAT)	Colon cancer	Inhibition of tumor growth	[91]

**Table 4:** Gal-3 inhibitors in tumor progression

Inhibitor	Target	Effect	Refs
Belapectin(GR-MD-02)	Sarcoma, breast and prostate cancer xenografts, musculoskeletal tumors, clinical trials in patients with metastatic melanoma and head and neck squamous cell carcinoma	Inhibition of tumor growth, restore the T cells surveillance	[92]
GCS-100	Multiple myeloma, diffuse large B-cell lymphoma cell lines, prostate cancer cell lines	Induction of apoptosis, inhibits cell proliferation and migration	[93]
TD139	Thyroid cancer cells	Induction of apoptosis, inhibits migration, invasion and resistance	[94]
GB1107	Thyroid cancer cells, gastric cancer, lung adenocarcinoma xenografts, prostate cancer	Inhibits tumor growth and metastasis, inhibits cell migration and invasion	[94]
Modified citrus pectin (MCP)	Urinary bladder cancer, prostatic cancer, colon cancer xenografts, ovarian cancer cells, breast cancer, renal cell carcinoma	Induction of apoptosis, inhibits proliferation, growth, invasion, migration, adhesion, and metastasis, enhances radiosensitivity	[91, 92, 95]
HH1-1	Pancreatic cancer	Suppress cell proliferation, arrest the cell cycle in S phase, induce cell apoptosis, inhibit angiogenesis and impede tumor cell migration and invasion	[96]
Curcumin	Glioblastoma cells	Reduces induction of UV-C radiation and alkylating agents	[97]
RN1	Pancreatic ductal adenocarcinoma	Inhibits cell growth	[95]

Thiobisgalactoside (TDG) is a synthetic disaccharide targeting Gal-1 [88]. Modified citrus pectin (MCP, PectaSol-C), RN1 and GM-CT-01 (DAVANAT) are natural polysaccharides evidenced to have galectin inhibiting activity, by which MCP and RN1 inhibit Gal-3 function, and GM-CT-01 has affinity for both Gal-1 and Gal-3 [91, 95]. A modified DAVANAT version, Gal-3 inhibitor GR-MD-02, was shown to be effective in treating mouse nonalcoholic steatohepatitis [92]. Novel Gal-3 inhibitor GCS-100, a polysaccharide derived from citrus pectin, exhibits potential effects in treatment for myeloma [93]. A synthetic peptide named Anginex specifically binds to Gal-1 and exerts antiangiogenic and antitumor effects [90]. The non-peptide compound OTX008 targets against Gal-1 and inhibits tumor cell proliferation, cell cycle, and angiogenesis [89]. Diphenyl sulfone (DBF) is a partial peptidomimetic agent and its derivative 6DB7 designed to inhibit Gal-1 also suppresses angiogenesis in tumor tissue [98]. Recently, a preclinical evaluation of GB1107 and TD139, inhibitors of Gal-3, revealed promising outcomes in the treatment and prevention of the metastatic spread of thyroid cancer [94]. HH1-1, a polysaccharide isolated and purified from safflower oil, blocks the interaction between Gal-3 and EGFR and inhibits the growth of pancreatic cancer cells [96]. Interestingly, curcumin, a well-known natural bioactive substance derived from the rhizome of the ginger family, also showed strong inhibitory activity against Gal-3 overexpression, implying the underlying use in oncologic applications [97].

Immediately following the preclinical advances in laboratories, a number of Gal-1 and Gal3 inhibitors have already been applied in clinical trials (Table 5). Most of the 13 trials (updated till Dec 2022) have employed either galectin inhibitors alone (e.g. GM-CT-01 and GR-MD-02) or in combination with chemotherapeutic agents (e.g. 5-fluorouracil) or monoclonal antibodies (e.g. ipilimumab or pembrolizumab) for treatment of solid tumors. In these trials, multicenter phase I or II trials have been initiated for various types of tumors, including non-small cell lung cancer, metastatic melanoma, squamous cell head and neck cancer, colorectal cancer, bile duct and gallbladder cancer, breast cancer, prostate cancer, metastatic melanoma and diffuse large B-cell lymphoma. So far, only two Phase I trials (NCT00054977 and NCT02117362) and one Phase II trial (NCT01681823) have been completed. Unfortunately, over half of the studies have been withdrawn or terminated due to poor treatment effects or lack of funding, suggesting that not all galectin inhibitors are suitable for all tumor types and that their efficacy may vary depending on the protocol design and the galectin expression profile in each individual.

**Table 5:** Completed and ongoing clinical trials targeting galectins in oncology

NCT Number	Intervention	Target	Condition	Phase	Status
NCT05240131	GB1211 combined with Atezolizumab	Gal-3	Non-small cell lung cancer	I/II	Recruiting
NCT01724320	OTX008	Gal-1	Solid tumors	I	Unknown
NCT02575404	GR-MD-02 combined with Pembrolizumab (anti-PD-L1)	Gal-3	Melanoma, non-small cell lung cancer, and squamous cell head and neck cancers	I	Active, not recruiting
NCT00388700	GM-CT-01 combined with 5-FU, Avastin and Leucovorin	Gal-1&3	Colorectal cancer	II	Withdrawn
NCT00110721	GM-CT-01 plus 5-FU	Gal-1&3	Colorectal cancer	II	Terminated
NCT00386516	GM-CT-01 combined with 5-FU	Gal-1&3	Bile duct cancer Gallbladder cancer	II	Withdrawn
NCT00054977	GM-CT-01 with and without 5-FU	Gal-1&3	Colorectal, lung, breast, head and neck, and prostate cancers	I	Completed
NCT04666688	LYT-200 alone or combined with Gemcitabine/nab-paclitaxel or Anti-PD-1	Gal-9	Metastatic cancer, solid tumor, cholangiocarcinoma	I/II	Recruiting
NCT04987996	GR-MD-02 plus Pembrolizumab or Pembrolizumab	Gal-3	Melanoma, head and neck squamous cell carcinoma	II	Suspended
NCT01681823	Dietary Supplement: PectaSol-C Modified Citrus Pectin (MCP)	Gal-3	Prostatic neoplasms	II	Completed
NCT01723813	Peptide vaccinations plus GM-CT-01	Gal-3	Metastatic melanoma	I/II	Terminated
NCT02117362	GR-MD-02 and Ipilimumab	Gal-3	Metastatic melanoma	I	Completed
NCT00776802	GCS-100 combined with Etoposide and Dexamethasone	Gal-3	Diffuse large B-cell lymphoma	I/II	Withdrawn

Data were collected from the NIH Clinical Trials website (https://clinicaltrials.gov/, till Dec 2022)

### Overview of autophagy

In all eukaryotic cells, autophagy is highly conserved event that cells degrade their damaged organelles and macromolecules through lysosomes to maintain normal biological homeostasis and basic cell activities. Essential roles of autophagy include protecting cells from damaged proteins, shielding cell organelles from toxins, maintaining cell metabolism and energy homeostasis, and promoting cell survival, particularly in stressful conditions (e.g., starvation, hypoxia, endoplasmic reticulum stress, infection, and lack of growth factors) [99–101]. Depending on the pathway of cellular material passaging to the lysosome, macroautophagy, microautophagy and chaperone-mediated autophagy are often classified [102]. Since macroautophagy is the primary autophagic process in eukaryotic cells, it is thereafter simply referred as autophagy.

### The autophagic pathway

**Fig. 2:**
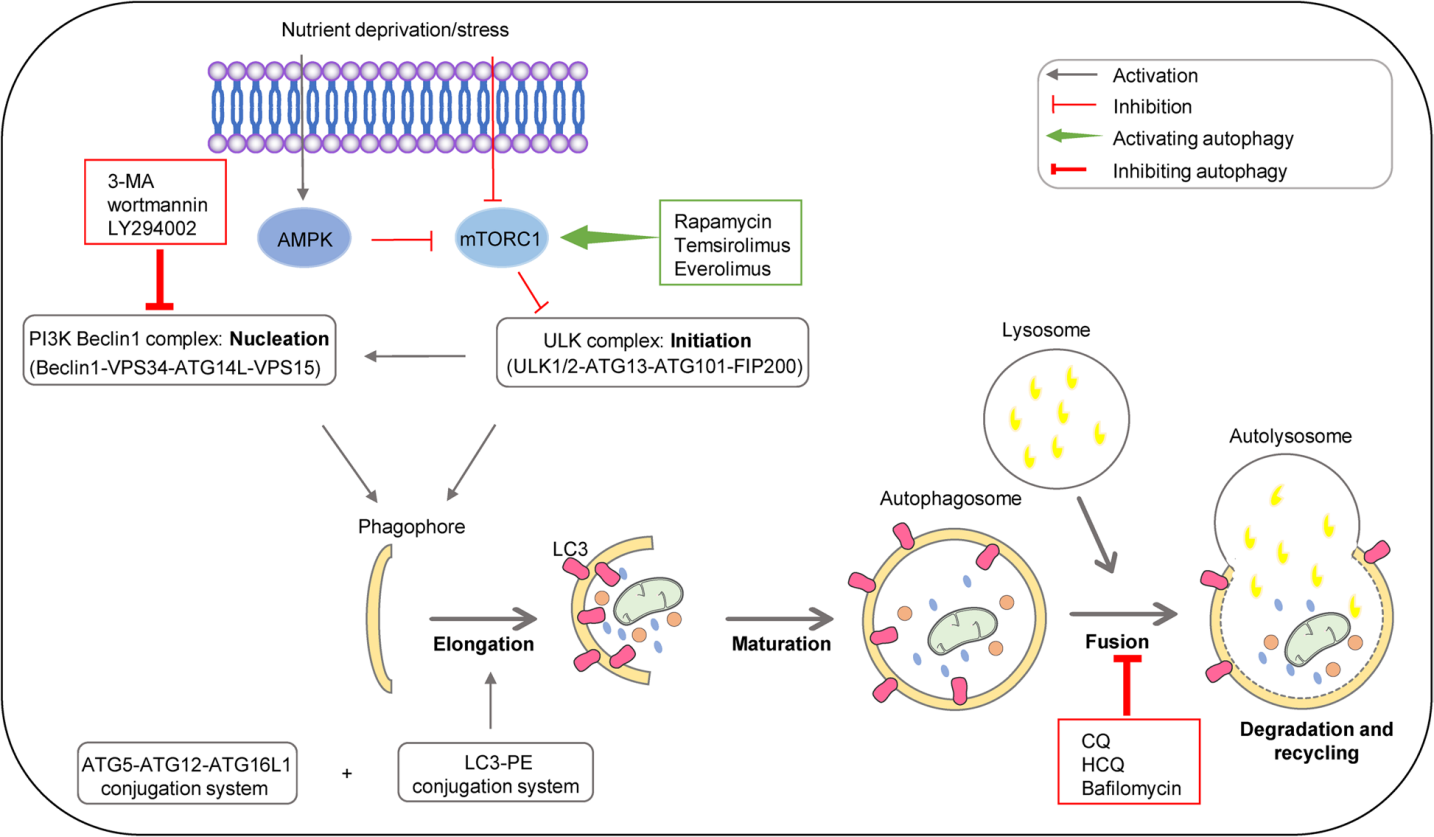
Schematic overview of the autophagy pathway and modulators. AMPK: adenosine monophosphate (AMP)-activated protein kinase; mTORC1: mammalian target of rapamycin complex 1; ULK: Unc-51-like kinase; ATG: autophagy-related gene; FIP200: focal adhesion kinase (FAK) family-interacting protein of 200 kDa; VPS34: vacuolar protein sorting 34; PI3K: phosphoinositide-3-kinase; LC3; PE: phosphatidylethanolamine

Autophagy is a complex multistep process involving more than 30 core autophagy-related (ATG) proteins. We here summarized the autophagic pathway briefly into six phases (Fig. 2.): initiation phase, precursor nucleation phase, extension phase, autophagosome maturation phase, autophagosome-lysosome fusion phase and cargo degradation and recycling phase.

In the initiation stage, the AMP-activated protein kinase (AMPK) and the mammalian target of rapamycin (mTOR) signaling pathways are the pivotal regulators [103, 104]. Activation of AMPK, a key cellular energy sensor, leads to the inactivation of mTOR pathway, and subsequently switches on autophagy. Once mTORC1 kinase activity is suppressed under situations such as nutrient deprivation or stress, AMPK and mTOR together target the Unc-51 like kinase (ULK) complex directly, which activates and initiates the formation of autophagosomes [105]. This is followed by the recruitment of other effector proteins through phosphorylation and activation of the phosphoinositide 3-kinase (PI3K) complex that mediates phagocytic vesicle nucleation [106]. After the formation of autophagic precursors, the phagocytic vesicle membrane continues to extend until it completely envelops the contents to be degraded. During this process, the implication of two ubiquitin-like protein modification systems, ATG12-ATG5 and LC3-PE, are the hallmark events [107, 108]. With the extension and closure of the autophagosome, mature autophagosomes are formed [109]. The progressive removal of ATG proteins from the outer membrane of autophagosomes allows the recruitment of proteins responsible for lysosomal delivery and proteins mediating the fusion with lysosomes [8]. Next, the autophagosomal outer membrane fuses with the lysosomal membrane to form autophagic lysosomes [110]. Finally, the autophagosomal endosomal membrane and contents are degraded into small molecules by acid hydrolases within the lysosome and the recovered nutrients are released back into the cytoplasm for reuse, i.e. the recycling phase [106].

### Role of autophagy in cancer

Autophagy has been characterized to regulate tumorigenesis, tumor metastasis, tumor stem cells, and treatment resistance [111]. Despite that a couple of autophagy modulators have been used to treat cancer, the dual role of autophagy, as tumor suppressor or promoter, has limited its use in clinical applications and is a major reason for treatment failure [111, 112]. Alternatively, regulation of autophagic flux may provide other options for autophagy-based therapy strategies. On top of that, inhibitors (Chloroquine, Hydroxychloroquine, GNS651 and Clarithromycin) or inducers (Rapamycin, Temsirolimus, Everolimus and Metformin) of autophagy can be applied for cancer treatment separately, depending on the respective cancers and the specific stage of tumorigenesis [113, 114].

#### Autophagy as tumor suppressor

Autophagy inhibition may lead to genomic instability and the aggregation of damaged organelles and proteins generated by tumor cells [115]. In this sense, autophagy is initially believed to inhibit tumor progression during the early stages of cancer development. Of note, EMT promotion caused by autophagy inhibition have been widely reported in cancer research and cancer therapy [116, 117]. Beclin1 is involved in the autophagosome formation and exerts an antitumor effect by promoting apoptosis in cancer cells, and deficiency in Beclin1 has been shown to promote tumor cell proliferation in several cancers (e.g. ovarian, breast and prostate cancers) [118, 119]. Moreover, Beclin1 expression has been shown to be downregulated in a variety of malignant tumors including glioblastoma, liver cancer, osteosarcoma, and cervical squamous carcinoma [120]. Some other ATG genes and proteins have also been suggested to be tumor suppressors since deletion of ATG5 and ATG7 induces the development of benign liver tumors in mice [121]. Interestingly, ATG7 conditional knockout mice developed multiple tumors in the liver, and additional knockdown of the autophagy receptor protein p62 reversed this phenotype, suggesting that p62 accumulation due to autophagy inhibition promotes tumor formation [121]. Tissue-specific knockdown of ATG5 or ATG7 also promotes lung cancer driven by oncogenic mutations [122]. In addition, overexpression of ULK2 induces glioblastoma autophagy and inhibits astrocyte transformation and tumor growth [123]. These studies suggest that autophagy plays a tumor-suppressive role in several tumors.

#### Autophagy as tumor promoter

In contrast, autophagy for a long time has been described as a pro-survival mechanism in most advanced tumors, by promoting tumor to adapt to different stressed conditions such as hypoxia or nutritional deficiency, thus mediating tumor progression [124]. The expression of hypoxia-inducible factor 1α (HIF1α) is induced under hypoxia, which mediates AMPK activation and mTOR inhibition, thus promoting autophagy and cell survival [125]. The autophagic cargo adapter p62 is also downregulated when hypoxia-induced autophagy occurs, leading to activation of Ras/ERK signaling and enhanced cell proliferation [126]. Moreover, the autophagy gene ATG5 is highly expressed in gastric and prostate cancer [127, 128], ATG7 is abundantly present in bladder cancer [129], and conditional knockout of autophagy gene FIP200 shows reduced mammary tumorigenesis [130]. Autophagy promotes EMT and metastasis of hepatocellular carcinoma and pancreatic tumor cell [131, 132], and it also facilitates the oncogenic RAS-mediated tumorigenesis, which together indicates the pro-tumorigenic role of autophagy [133]. Apart from the role of promoting tumor cell proliferation, autophagy also participates in tumor metastasis and stemness maintenance of cancer stem cells (CSCs), and the autophagic fluxes were enhanced in specific types of CSCs [134]. Taken together, autophagy can also promote tumorigenesis and tumor progression, implying that autophagy inhibition may also be conducive in the treatment for particular cancers.

#### Targeting autophagy in cancer therapy

As mentioned above, due to the dual role of autophagy in tumorigenesis, both autophagy activators and inhibitors have been used in preclinical and clinical studies (Fig. 2.). Typical autophagy-inducers include classic mTOR1 inhibitors such as Rapamycin (RAPA, Sirolimus), RAPA analogs Temsirolimus (CCI-779) and Everolimus (RAD001), all of which have been used to treat different cancers [114]. Several autophagy-stimulatory compounds have also been identified to show anticancer effects, including metformin that upregulates AMPK, resveratrol that activates silent information regulator 1 (SIRT1), and perifosine that inhibits AKT/mTOR signaling [135]. Although induction of cytoprotective autophagy in early-stage tumors may inhibit cancer progression, enhanced autophagy resulting from chemotherapy and radiotherapy has been shown to contribute to therapeutic resistance in several cancers [111]. In a number of advanced tumors, increased autophagic activity promotes the survival of tumor cells, thus inhibition of autophagy may enable the blockade of tumor progression. In this context, majorities of current anticancer strategies have been inclined to achieve autophagy inhibition or to combine autophagy inhibition with other treatment approaches.

Given the complexities of the autophagic process, various autophagy inhibitors targeting different stages of the autophagic process have been established. Wortmannin, 3-methyladenine (3-MA), and LY294002 block autophagosome formation by targeting class III PI3K at the autophagy initiation stage, and chloroquine (CQ), hydroxychloroquine (HCQ), monensin, and bafilomycin A1 are able to inhibit autophagy by preventing the fusion of autophagosomes and lysosomes [136]. Among these inhibitors, only CQ and HCQ are currently available for the clinical use [136]. Facing the immediate demand in clinical cancer therapy, several new inhibitors of autophagy are being tested in preclinical trials, with 3-MA, SAR405, Lys05, ROC-325, Spautin-1, MM124 and MM137 being most promising [114, 137]. These compounds are targeting different autophagic process, and further studies are needed to determine their clinical value in cancer treatment. Apart from drugs that targeting autophagic process directly, other approaches including microRNA, nanoparticles, natural products like luteolin, also exert autophagy-regulation and anti-tumor effects [138–140]. Since the roles of autophagy are bidirectional and may vary in distinct cancer types, the uses of autophagy inducers or inhibitors should be customized in the future cancer treatments.

### Galectin and autophagy

#### Galectin regulation of autophagy

**Fig. 3:**
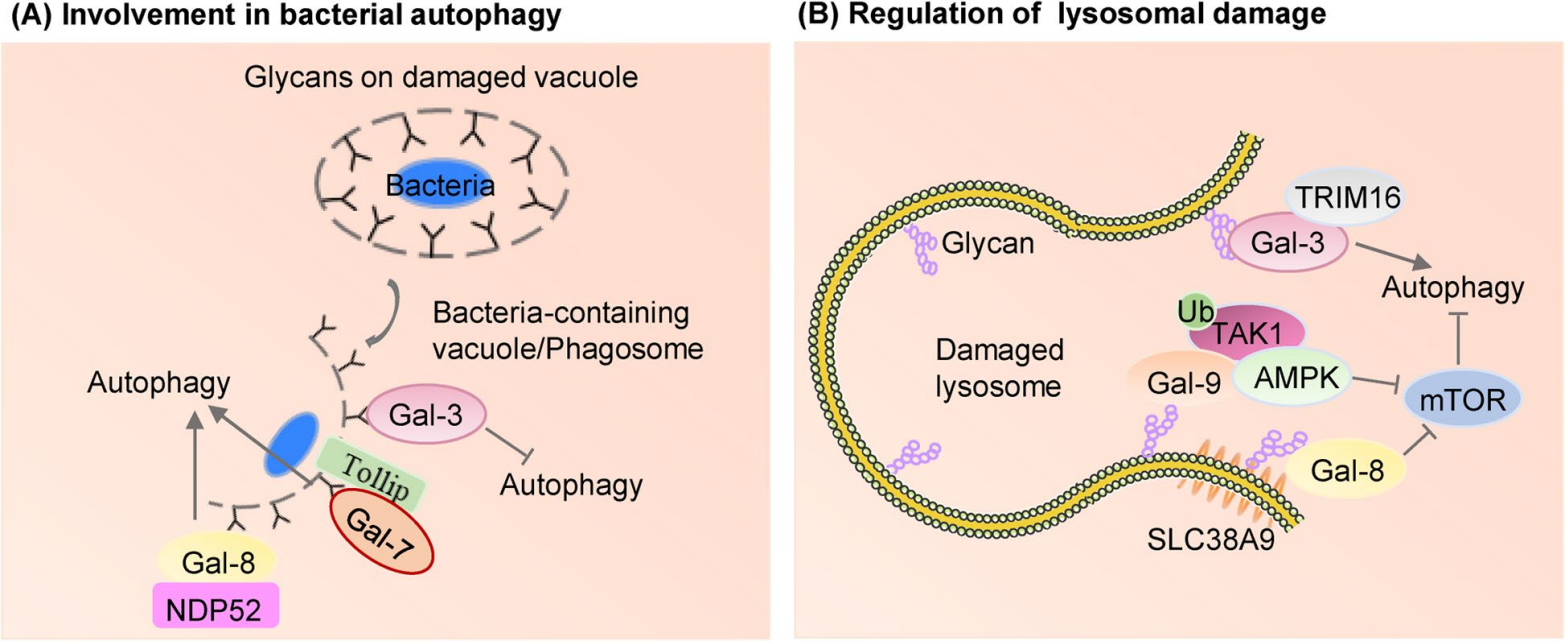
Roles of galectins in autophagy. **A** Gal-8 promotes antibacterial autophagy by recognizing host glycans on ruptured vacuolar membranes and interacting with the autophagy adaptor protein NDP52. Autophagy receptor Tollip facilitates bacterial autophagy by recruiting Gal-7 in Response to bacterial Infection. Gal-3 accumulates at damaged phagosomes containing bacteria leading to a stronger anti-autophagic response. **B** Galectins recognize membrane damage by binding to lumenal glycans upon their exposure to the cytosol following endomembrane damage. During lysosomal damage, Gal-8 interacts with SLC38A9-Ragulator-mTOR complex and inhibits mTOR activity thereby inducing autophagy, whereas Gal-9 activates AMPK in response to lysosomal injury by promoting ubiquitination of TAK1. TRIM16 interacts with Gal-3 in response to damaged endomembrane

A growing body of evidence supports the relationship between galectins and autophagy. A seminal work in 2012 reported by Teresa Thurston revealed the significance of Gal-8 in autophagy, which has thereafter drawn more attention to Gal-autophagy interaction. Under physiological conditions, Gal-8 protects cells from bacterial invasion by regulating the integrity of endosomes and lysosomes and recruiting the autophagic cargo receptor NDP52 to activate antibacterial autophagy [141, 142]. Similarly, the galectin-mediated protective mechanisms also play a role in resistance to the invasion of viruses [143]. By specifically interacting with the selective autophagy adaptor TAX1BP1, Gal-8 enables macrophages to effectively target mycobacterium tuberculosis for selective autophagy [144]. This finding suggests that Gal-8 may serve as a potential target for the treatment of tuberculosis. In addition, Gal-7 interacts with the bacterial autophagy receptor Tollip and also regulates bacterial autophagy along with other autophagy receptors that are recruited [145]. In contrast to Gal-7 and Gal-8, cellular accumulation of Gal-3 leads to a strong anti-autophagic response, by sensing glycosylation changes on cell surface and modulating cellular responses through differential recognition of glycans on ruptured phagosomal membranes [146] (Fig. 3A.).

Galectins also modulate autophagy in processing with endosomal damage associated with lysosomal dysfunction (Fig. 3B.). In response to damaged endosomes, TRIM16 and Gal-3 interact in a ULK1-dependent way to mobilize the key autophagy regulators ATG16, ULK1 and Beclin1, and activate selective autophagy [147, 148]. Lysosomal damage is one of the strongest stimulators of autophagy, but how lysosomal membrane damage activates autophagy is not fully understood. It has been shown that, in response to lysosomal damage, Gal-8 inhibits mTOR activity through Ragulator-Rag signaling, while Gal-9 activates AMPK in a Gal-9-ubiquitination dependent manner to induce autophagy [149–151].

#### Galectin-autophagy association in cancer

**Fig. 4:**
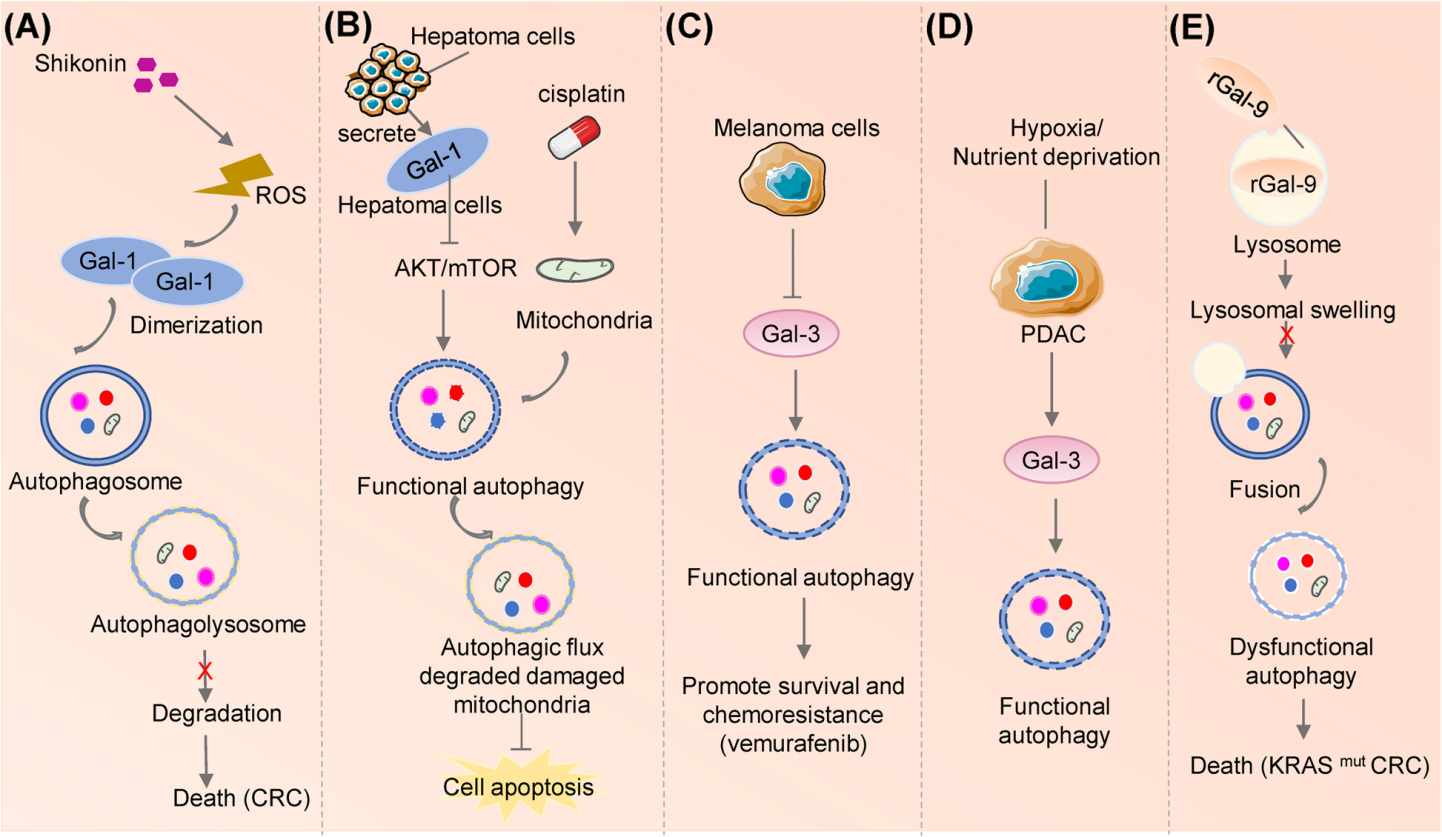
Galectins are involved in autophagy regulation in tumors. **A** Shikonin induces ROS accumulation and dimerization of Gal-1 in human colorectal cancer cells by inhibiting the degradation of autophagolysosomes. **B** In hepatoma microenvironment, secreted Gal-1 binds to hepatoma cells and triggers autophagic flux via inhibiting AKT-mTOR activities. Gal-1-induced autophagy can target damaged mitochondria to reduce both mitochondrial membrane potential loss and cell death under cisplatin treatment. **C** Gal-3 acts as a negative regulator of autophagy in melanoma cells. Gal-3 inhibition increases autolysosome formation and resistance of melanoma cells to vemurafenib. **D** Gal-3 is upregulated under nutrient deprivation and hypoxia in pancreatic cancer which enhances the autophagic flux in tumor cells. **E** In KRAS^mut^ colorectal cancer (CRC), rLGALS9 acts as a lysosomal inhibitor and inhibits autophagosome-lysosome fusion, leading to autophagosome accumulation, excessive lysosomal swelling and cell death

In contrast to extensive studies on galectin or autophagy alone, the function of Galectin-regulated autophagy in tumor biology has been less elucidated. Shikonin induces reactive oxygen species (ROS) accumulation in colorectal cancer cells, which promotes Gal-1 expression and dimerization and suppresses autophagic flux by blocking the degradation of autophagolysosomes, and eventually stimulates autophagic cell death [152]. In addition, Gal-1 has been reported to promotes resistance to radiotherapy and chemotherapy of tumor cells (Fig. 4.), and downregulation of Gal-1 could suppress autophagy and enhance the sensitivity of neuroblastoma to cisplatin treatment [153]. Similarly, Gal-1 secreted by hepatoma cells causes autophagic flux by blocking AKT-mTOR activities in an autocrine manner. Autophagy induced by Gal-1 alleviates the damage of mitochondrial membranes and cell death under cisplatin treatment, which results in cisplatin resistance. Therefore, Gal-1 inhibition by thiobisgalactoside significantly increases the senstivity of cisplatin in hepatocellular carcinoma treatment [154]. Notably, knockdown of Gal-1 also increased the sensitivity of malignant melanoma and glioblastoma to temozolomide, yet without inducing apoptosis or autophagy [155, 156].

Gal-3 may negatively regulates autophagy in melanoma cells, which is evidence by reduced the pro-survival function autophagy and consequent treatment resistance [157]. In pancreatic cell lines, Gal-3 is involved in the regulation of autophagy to maintain intracellular homeostasis under extreme conditions such as hypoxia and nutrient deficiency [158].

Recombinant Galectin-9 (rGal-9), through its internalization and accumulation in lysosomes, causes lysosome swelling and inhibited fusion of autophagosome and lysosomes, leading to boosted cell death in KRAS-mutant colon cancer [159].

Gal-8 has recently been suggested as a novel biomarker for autophagy-related tumors in glioblastoma (GBM) [160]. However, to our knowledge, whether Gal-8 regulates tumor growth through autophagy-dependent mechanisms is unknown. Our group found that Gal-8 expression correlates with the progression of GBM and that hypoxia induces GBM autophagy to promote tumorigenesis through Gal-8 (data unpublished). Our data demonstrate that inhibiting Gal-8 to further block autophagy in hypoxic microenvironment could attenuate the cell growth in GBM both in vitro and in vivo.

## Conclusions and Outlook

Half a century ago, galectins were discovered as a glycan-binding protein. We now have known better about this family as more physiological and pathological functions of galectins are gradually revealed. Considering the broad roles of galectins in tumorigenesis, the trend of galectins being potential diagnostic markers and therapeutic targets in cancers has been well recognized. Here in this review, we summarize the roles of galectins in normal and cancerous cells, highlight the clinical applications of galectin inhibitors in cancer research, and introduce autophagy as a vital mediator in galectin regulated tumorigenesis, aiming to shed lights on galectin-autophagy targeted anticancer strategies.

There are still a number of concerns when galectins-targeted cancer treatments are to be applied into human patients. Most preclinical research is conducted in cultured cells and animal models, which are not able to fully recapitulate the complex environments within human tumors, in addition to the high heterogeneity across tumor types. Even though more than a dozen of clinical trials has started, unfortunately, failures and inclusive results seem inevitable. Furthermore, tumor cells express more than one galectin, and factors such as subcellular localization and posttranscriptional modifications of galectins add extra complexity onto the specificity of galectin in tumors. Therefore, tumor-specific galectin-targeting is crucial before therapy commences. Another aspect to take into account is that the cancer stem cells hidden in the solid tumor, may show distinct galectin expression profiles and sensitivity to galectin intervention compared to general tumor cells, providing the strategic possibility of targeting the ‘ancestral’ cells to block potential malignancy in their successor tumor cells. Additionally, increasing evidence are unveiling the intracellular roles of galectins in either mediating signaling transduction initiated from outer space or maintaining cell hemostasis. Intracellular galectins are to be particularly targeted for drug design other than being neglected, since the majority of current inhibitors are designed to block the extracellular functions of galectins.

Targeting the autophagic pathway in anticancer therapies also appears to be promising, although not many autophagy-based compounds are currently in use for treating cancer. As expected, the dual role of autophagy leads to the decision whether autophagy in tumor cells should be inhibited or induced hard to make, and specific research is warranted to determine which types of cancer patients would benefit from autophagy-based strategies, considering our current knowledge that potential molecular mechanisms and specific targets of autophagy very likely diverge in different cancers. While the notion has been being more and more accepted that current clinical trials are meant to evaluate inhibition of autophagy alone or in combination with other treatments, encouragingly, a large body of evidence has shown that galectins are key autophagy inducers, suggesting that therapies targeting galectins in combination with other modulators such as autophagy in tumor progression may open new window to halt cancer progression.

Together, as galectin-based preclinical and clinical studies are emerging in cancer treatment, more applicable signature molecules of galectins as biomarkers for diagnosis, galectin-targeting drug design, cancer-specific drug delivery, in conjunction with combinations of autophagy-targeted agents, probably could provide better strategies to achieve precise dual-targeting in specific cancer type and various stages of tumorigenesis. These complexities and challenges are assumed to equally be exploited for the early diagnosis and successful treatments for cancers.

## Data Availability

Not applicable.

## Abbreviations

Gals: Galectins
CRDs: Carbohydrate recognition domains
EBV: Epstein-Barr virus
Po66-CBP: Po66 carbohydrate-binding protein
EMT: Epithelial-mesenchymal transformation
FAK: Focal adhesion kinase
ALCAM: Activated leukocyte cell adhesion molecule
Tim-3: T-cell immunoglobulin and mucin structural domain protein 3
PD-L1: Programmed cell death ligand 1
CRISPR: Clustered regularly interspaced short palindromic repeats
TDG: Thiobisgalactoside
MCP: Modified citrus pectin
DBF: Diphenyl sulfone
ATG: Autophagy-related
AMPK: AMP-activated protein kinase
mTOR: Mammalian target of rapamycin
ULK: Unc-51 like kinase
PI3K: Phosphoinositide 3-kinase
HIF1α: Hypoxia-inducible factor 1α
CSCs: Cancer stem cells
SIRT1: Silent information regulator 1
3-MA: 3-Methyladenine
CQ: Chloroquine
HCQ: Hydroxychloroquine
ROS: Reactive oxygen species
rGal-9: Recombinant Galectin-9
GBM: Glioblastoma
